# Cerdulatinib, a novel dual SYK/JAK kinase inhibitor, has broad anti-tumor activity in both ABC and GCB types of diffuse large B cell lymphoma

**DOI:** 10.18632/oncotarget.6316

**Published:** 2015-11-05

**Authors:** Jiao Ma, Wei Xing, Greg Coffey, Karen Dresser, Kellie Lu, Ailin Guo, Gordana Raca, Anjali Pandey, Pamela Conley, Hongbo Yu, Y. Lynn Wang

**Affiliations:** ^1^ Department of Pathology and Laboratory Medicine, Weill Cornell Medical College, New York, NY, USA; ^2^ Department of Pathology, University of Massachusetts Memorial Medical Center and Medical School, Worcester, MA, USA; ^3^ Department of Biology, Portola Pharmaceuticals, Inc., South San Francisco, CA, USA; ^4^ University of Chicago Laboratory School, Chicago, IL, USA; ^5^ Department of Pathology, Division of Genomic and Molecular Pathology, University of Chicago, Chicago, IL, USA; ^6^ Department of Medicine, University of Chicago, IL, USA

**Keywords:** diffuse large B cell lymphoma, cerdulatinib, SYK, JAK-STAT, molecularly targeted therapy

## Abstract

B-cell receptor (BCR) and JAK/STAT pathways play critical roles in diffuse large B-cell lymphoma (DLBCL). Herein, we investigated the anti-lymphoma activity of cerdulatinib, a novel compound that dually targets SYK and JAK/STAT pathways. On a tissue microarray of 62 primary DLBCL tumors, 58% expressed either phosphorylated SYK or STAT3 or both. SYK and STAT3 are also phosphorylated in a panel of eleven DLBCL cell lines although ABC and GCB subtypes exhibited different JAK/STAT and BCR signaling profiles. In both ABC and GCB cell lines, cerdulatinib induced apoptosis that was associated with caspase-3 and PARP cleavage. The compound also blocked G1/S transition and caused cell cycle arrest, accompanied by inhibition of RB phosphorylation and down-regulation of cyclin E. Phosphorylation of BCR components and STAT3 was sensitive to cerdulatinib in both ABC and GCB cell lines under stimulated conditions. Importantly, JAK/STAT and BCR signaling can be blocked by cerdulatinib in primary GCB and non-GCB DLBCL tumor cells that were accompanied by cell death. Our work provides mechanistic insights into the actions of cerdulatinib, suggesting that the drug has a broad anti-tumor activity in both ABC and GCB DLBCL, at least in part by inhibiting SYK and JAK pathways.

## INTRODUCTION

Diffuse large B-cell lymphoma (DLBCL) is the most common type of non-Hodgkin lymphoma (NHL) and accounts for approximately 40% of all NHL cases. The tumor progresses rapidly and treatment is normally initiated immediately after a patient is diagnosed with the disease. The standard chemoimmunoregimen, rituximab, cyclophosphamide, doxorubicin, vincristine and prednisone (R-CHOP), is effective in ~60% of patients, but nearly 50% of patients treated with R-CHOP will eventually progress and relapse. The death rate of DLBCL remains at approximately 30%. Thus, there is an urgent demand for the development of more effective therapies based on the understanding of molecular pathogenesis.

Aberrant B-cell receptor (BCR) signaling is implicated in B-cell malignancies including DLBCL. The BCR complex consists of surface immunoglobulins (sIg) that bind antigen in association with disulfide-linked heterodimer CD79A and CD79B proteins [1]. Upon antigen binding, the conformational change of sIg transduces the signal to the cytoplasmic portions of CD79A/B and results in the phosphorylation of the immunoreceptor tyrosine-based activation motif (ITAM) by SRC-family protein tyrosine kinase LYN. Phosphorylation of ITAMs then recruits cytosolic tyrosine kinase SYK and causes its phosphorylation and activation. SYK then triggers activation of the PI3K-AKT and BTK-PLCγ2 pathways with the subsequent generation of inositol triphosphate (IP_3_) and diacylglycerol (DAG). This event is followed by activation of multiple distal signaling pathways for B-cell activation, such as RAS-MAPK pathway, PKC activation and formation of CARD11/BCL10/MALT1 complex, and subsequent NFκB activation [1].

The first evidence that dysregulated BCR activation is a major contributor to DLBCL pathogenesis came from gene expression profiling analysis. Based on relatedness of gene expression profiles to normal B-cell subsets, DLBCL were classified into three cell-of-origin subtypes: germinal center B-cell (GCB) subtype, activated B-cell (ABC) subtype and primary mediastinal B-cell lymphoma (PMBL) [2–5].

Subsequent studies revealed that different signaling pathways are involved in these distinct subtypes of DLBCL [6]. The main molecular and genetic abnormalities in GCB DLBCL include activation of PI3K/AKT/mTOR pathway, BCL2 translocations, and BCL6 rearrangements and overexpression, MYC rearrangements, and EZH2 mutations; while ABC DLBCL is featured with the activation of BCR, NF-κB and JAK-STAT pathways with associated mutations in genes including *CD79A/B, CARD11, TNFAIP3 (A20)* and *MYD88*. For PMBL DLBCL, key molecular abnormalities include CIITA translocations, amplification of REL, amplification of chromosome region 9p24 containing PD-L1, PD-L2 and JAK2 loci, and activation of NF-κB pathways [3, 7, 8]. These molecular subtypes are clinically relevant as patient outcomes and responses to chemoimmunotherapeutic regimens are different: GCB DLBCL has much higher response rate than ABC subtype to R-CHOP, while most of PMBL DLBCL can be cured with DA-EPOCH-R regimen [6].

Additional information regarding the molecular features of DLBCL was revealed by analysis of gene expression profiles with consensus clustering which groups lymphomas by functional relatedness of genes [9]. This analysis also identified three groups: BCR/proliferation, OxPhos, and Host response. The BCR group is featured with high expression of SYK mRNA along with mRNA of other BCR pathway components. Concordance between the cell-of-origin and consensus clustering schemes, however, is poor. A common molecular feature identified is the increased BCR signaling in a significant fraction of DLBCL patients, ABC-DLBCL by cell-of-origin scheme and BCR-DLBCL by consensus clustering scheme. These studies provide a strong rationale to target BCR signaling in DLBCL.

Previously, several groups including ours have explored the potential of inhibiting BCR pathways in DLBCL cell lines and primary tumor cells [10–14]. We showed that targeting LYN [12] or SYK [13] inhibits BCR signaling and cell proliferation in a subset of DLBCL. Shipp's group further demonstrated the cells responding to SYK inhibition carry the BCR signature with either high or low NFκB activity [14].

From a clinical perspective, targeting the BCR pathway, however, has met with limited success in DLBCL patients compared to patients with other types of NHL and chronic lymphocytic leukemia (CLL). Fostamatinib, a SYK inhibitor, produced an objective response in 5 of 23 (22%) relapsed/refractory (R/R) DLBCL patients in a phase I/II study [15]. Enzastaurin, a PKCβ inhibitor, produced 3 complete responses and 1 stable disease in 55 R/R patients [16]. As to ibrutinib, a BTK inhibitor, in a phase II study of 80 R/R DLBCL, only 37% ABC and 5% GCB subtypes were responsive and the response rate of all DLBCL tumors was 19% [17]. Genetic analysis revealed that mutation in MYD88 can nullify the effect of BCR signaling blockade via Toll-like-receptor (TLR) signaling pathway leading to downstream NF-κB activation [17]. In addition, gain-of-function mutations in downstream CARD11 and A20 can drive the constitutive activation of the NF-κB pathway irrespective of upstream BTK blockade. The complexity of molecular and genomic alterations in DLBCL has severely limited the effectiveness of single-targeted therapy, thus the combination of targeted agents or multi-targeted agents have greater appeals in improving treatment response in DLBCL.

The Janus kinase and Signal Transducer and Activator of Transcription (JAK-STAT) pathway represents another important signaling pathway in the pathogenesis of DLBCL. One mechanism of STAT3 activation in ABC DLBCL (or non-GCB) has been defined. STAT3 is both overexpressed and activated primarily as a function of autocrine secretion of IL-6/IL-10 by tumor cells [18], a survival mechanism that can be promoted in the context of mutations that drive NF-κB activation and subsequent NF-κB-mediated cytokine expression [19]. In addition, it is possible that non-tumor cells of the tumor microenvironment promote malignant cell survival in part via paracrine cytokine secretion [20, 21]. Clinically, STAT3 activation, as reflected by STAT3 phosphorylation has been associated with worse survival in patients treated with R-CHOP [22]. *In vitro* inhibition of STAT3 activity with either JAK inhibitors or STAT3 knockdown results in decreased cell proliferation and increased apoptosis in ABC tumor cell lines [18, 23]. Moreover, early clinical studies suggest that targeting JAK/STAT pathways using small molecule JAK inhibition [24], STAT3 knock down (Hong DS, et al. 2013 ASCO annual meeting abstract #8523), or a neutralizing antibody specific for IL-6 [25] may be beneficial for patients with B-cell malignancies.

Thus, literature evidence provides a strong rationale to target both BCR and JAK-STAT pathway in DLBCL. Cerdulatinib (previously known as PRT062070) is a novel orally available small-molecule ATP-competitive inhibitor that demonstrates inhibition of SYK, JAK1, JAK2, JAK3, and TYK2 in a biochemical assay [26] (Table 1). However, at the cellular level, cerdulatinib demonstrates specificity towards JAK1/JAK3 and TYK2, but not JAK2-mediated responses. The specificity of cerdulatinib was also demonstrated by its lack of inhibition of T cell receptor signaling or protein kinase C signaling in whole blood [26]. In animal models, the agent reduces inflammation in a rat model of autoimmune disease, and blocks B-cell activation and alleviates splenomegaly induced by chronic BCR stimulation in mice [26]. Notably, in primary CLL cells with the BTK^C481S^ mutation, cerdulatinib is able to overcome ibrutinib resistance by completely blocking the proliferation of the resistant cells [27–29]. Cerdulatinib is currently under investigation as a single orally administered agent in a dose escalation study in relapsed/refractory CLL and B cell non-Hodgkin lymphoma (NHL; NCT01994382). Initial clinical results have demonstrated good tolerability, significant inhibition of SYK and JAK, and greater than 50% target tumor reductions in patients with CLL and NHL (Flinn I, et al. 2015 ASCO annual meeting Abstract #8531). Herein, we further characterize antitumor activities of cerdulatinib in subtypes of DLBCL cell lines and primary tumor cells. The results suggest cerdulatinib exerts broad anti-tumor activity in both ABC and GCB DLBCL including cells with resistance to BCR-targeted therapy.

**Table 1:** Activity of cerdulatinib against selected kinases, and their expression in normal LN and lymphoma tissues

Kinase	IC50 (nM)	Expression in normal lymph node*	Expression in primary lymphoma tissue*
TYK2	0.5	ND	Mostly ND
MST1	4	ND	Mostly ND
ARK5	4	ND-Low	ND
MLK1	5	ND	ND
FMS	5	N/A	N/A
**AMPK**	**6**	**Medium**	**Medium to High**
**JAK2**	**6**	**Medium to High**	**Mostly High**
JAK3	8	N/A	N/A
**TBK1**	**10**	**Low to Medium**	**Low to Medium**
MARK1	10	Low to Medium	ND
**JAK1**	**12**	**Medium**	**Low to Medium**
PAR1B-α	13	N/A	N/A
TSSK	14	N/A	N/A
MST2	15	ND	ND
GCK	18	Low	Mostly Low
JNK3	18	ND	ND
RSK2	20	N/A	N/A
**RSK4**	**28**	**Medium to High**	**Mostly Medium**
**SYK**	**32**	**High**	**Mostly High**
CHK1	42	ND	ND
FLT4	51	N/A	N/A
**FLT3**	**90**	**Medium to High**	**Mostly High**
RET	105	Low to Medium	Mostly ND
ITK	194	N/A	N/A

ND: Not detected. N/A: Not Available.

* Tissue microarray data adapted from the human protein atlas website at: http://www.proteinatlas.org. IC_50_ data were derived from Coffey G, et al. [26].

## RESULTS

### STAT3 and SYK are active in an array of primary DLBCL tissues of both GCB and non-GCB subtypes

**Figure 1:**
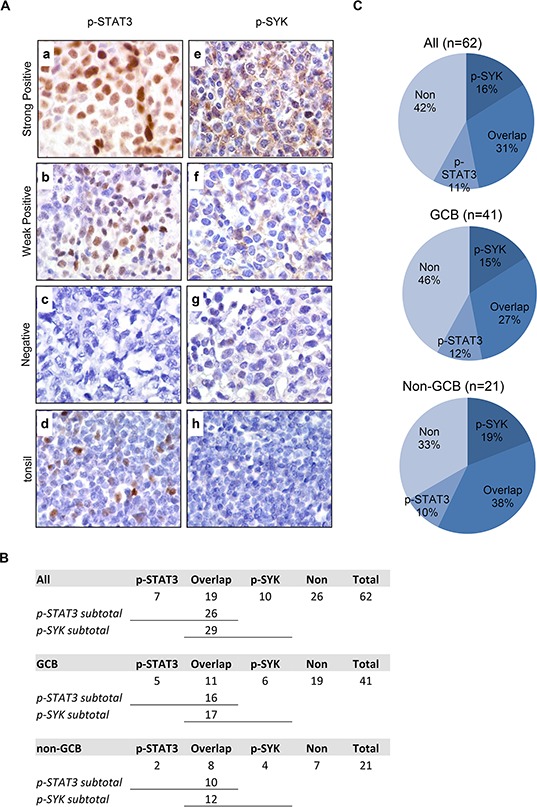
Expression of p-STAT3 (Y705) and p-SYK (Y525/526) in normal tonsil and primary DLBCL tissues on tissue microarray **A.** Expression of p-STAT3 (a-d) and p-SYK (e-h) was examined by immunohistochemistry on paraffin-embedded sections from DLBCLs (a-c and e-g) and normal tonsil tissue (d and h). **a.** A representative example of strongly positive p-STAT3 staining (60–90% lymphoma cells). **b.** A representative example of weakly positive p-STAT3 staining (30–50% lymphoma cells). **c.** A representative example of negative p-STAT3 staining (<30% lymphoma cells). **d.** Basal level of p-STAT3 expression in normal tonsil tissue with scattered positive cells in germinal center. **e.** A representative example of strongly positive p-SYK staining (60–90% lymphoma cells). **f.** A representative example of weakly positive p-SYK staining (30–50% lymphoma cells). **g.** A representative example of negative p-SYK staining (<30% lymphoma cells). **h.** Absence of p-SYK positivity in normal tonsil tissue. (a-h, x1,000). **B.** Table showing the number breakdown of p-SYK and p-STAT3 staining in all, GCB or non-GCB cases. **C.** Pie charts showing the percent breakdown of p-SYK and p-STAT3 staining in all, GCB or non-GCB cases.

To determine whether simultaneous targeting of both JAK/STAT and SYK is relevant in DLBCL, we examined the expression of p-STAT3 (Y705) and p-SYK (Y525/526) on a tissue microarray of 62 DLBCL primary tumors, including 41 germinal center-like (GCB) and 21 non-germinal-center-like (non-GCB) tumors classified using Han's algorithm [30] (Figure 1). p-STAT3 exhibits a characteristic nuclear staining pattern in DLBCL cases (Figure 1A). Patterns other than nuclear were excluded as positive staining. p-STAT3 staining in tonsil is included as control (Figure 1 A-d). A total of 26 (26/62, 42%) stained positive for nuclear p-STAT3; 16 were GCB type (16/41, 39%) and 10 were non-GCB type (10/21, 48%, Figure 1B). p-SYK expression was detected in 29 (29/62, 47%) cases with a characteristic peri-membrane staining pattern (Figures 1A and 1B). Patterns other than peri-membrane were excluded as positive staining. p-SYK staining in tonsil is included as control (Figure 1A-h). While occasionally germinal centers were found to contain a few scattered p-SYK positive cells, most of the germinal centers in the tonsil are completely negative for p-SYK. Of these 29 p-SYK positive cases, 17 were GCB type (17/41, 41%) and 12 were non-GCB type (12/21, 57%, Figure 1B). Interestingly, there are 19 cases (19/62, 31%) among the total 62 cases with reactivity for both p-SYK and p-STAT3, of which, 11 were GCB type (11/41, 27%) and 8 were non-GCB type (8/21, 38%, See Figure 1B for case numbers and Figure 1C for percentage breakdown). These data are consistent with the single stain results published previously on p-STAT3 by Huang et al [22] and on p-SYK by our group [13]. Together, these findings demonstrated that SYK and STAT3 are active in a significant number of DLBCL cases (Figure 1C).

### STAT3 and SYK are active in a panel of DLBCL cell lines of both GCB and ABC subtypes

**Figure 2:**
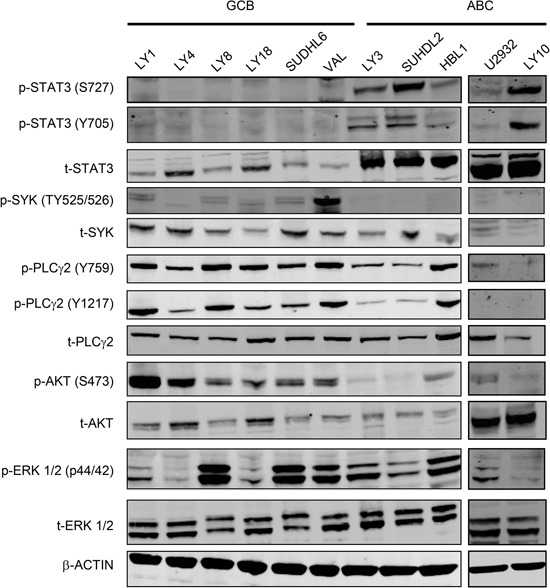
Distinct signaling pathways in GCB-DLBCL vs ABC-DLBCL cell lines Western blotting analysis of the basal levels of total and phosphorylated STAT3, SYK, PLCγ2, AKT and ERK in GCB and ABC DLBCL cell lines. Phosphorylated residues for each protein are indicated. β-actin was included as a loading control.

We then determined the basal expression levels of BCR signaling and JAK/STAT signaling molecules and their phosphorylation status in a panel of GCB and ABC DLBCL cell lines using immunoblotting analysis. The cell lines studied include six GCB cell lines: OCI-LY1 (LY1), OCI-LY4 (LY4), OCI-LY8 (LY8), OCI-LY18 (LY18), SUDHL6 (DHL6) and VAL, and 5 ABC cell lines: OCI-LY3 (LY3), SUDHL2 (DHL2), HBL1, U2932 and OCI-LY10 (LY10). As shown in Figure 2, three of the five ABC cell lines exhibited very high levels of total and phosphorylated STAT3 at both Y705 and S727 sites (LY3, DHL2 and LY10). In contrast, little total or phosphorylated STAT3 proteins were detected in the six GCB cell lines. The immunoblotting also revealed some interesting findings regarding all the GCB and HBL1 cell lines. Although the expression and phosphorylation of SYK, PLCγ2 and ERK and total AKT levels are variable among the cell lines, in general, all GCB appear to express higher levels of p-SYK, p-PLCγ2 (at both Y759 and Y1217) and p-AKT suggesting downstream BCR activity is more active in these cell lines. Among five ABC cell lines, HBL1 cell line appears to be the only one that expresses high levels of p-PLCγ2 (Figure 2). Taken together with Figure 1, active forms of SYK and JAK are expressed in a wider range of DLBCL tumors than each of the single molecule alone.

### Both ABC and GCB subtypes of DLBCL are sensitive to dual SYK/JAK inhibition with cerdulatinib

Cerdulatinib is an orally available ATP-competitive small molecule kinase inhibitor currently in clinical development for the treatment of B-cell malignancies (NCT01994382). Early clinical results (Flinn I, et al. 2014 ASCO annual meeting Abstract #2619) suggest that high level of SYK and JAK inhibition is achieved in treated patients, with an acceptable safety profile. In biochemical assays, cerdulatinib demonstrated inhibitory activity against 24 kinases with IC_50_'s < 200 nM (Table 1). Most of the kinases affected by cerdulatinib are not expressed at all, or expressed at very low levels in normal lymph nodes and primary lymphoma tissues. Seven of these affected kinases are notably expressed including AMPK, JAK2, TBK1, JAK1, RSK4, SYK, and FLT3 (Bold in Table 1). Cerdulatinib inhibits SYK and JAK1 with IC_50_'s of 32 nM and 12 nM, respectively. A recent publication detailing the translation of biochemical assays to cellular potency and selectivity revealed that cerdulatinib principally acted as a SYK, JAK1/3, and TYK2 inhibitor, lacking cellular activity against JAK2, against the SRC family members LCK and LYN, as well as the structural homolog ZAP70 [26]. Potency against AMPK, TBK1, RSK family members, and FLT3 has not been determined in cellular assays.

**Figure 3:**
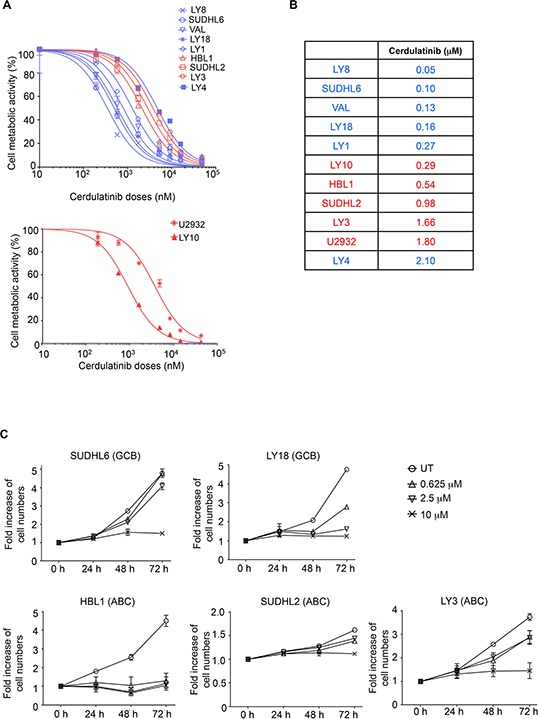
Both ABC and GCB subtypes of DLBCL are sensitive to dual SYK/JAK inhibition with cerdulatinib **A.** DLBCL cell lines were treated with various concentrations of cerdulatinib for 72 h followed by MTT assay. GCB are highlighted in blue and ABC in red. **B.** IC_50_ values were calculated using GraphPad Prism 5 software (GraphPad, La Jolla, CA). **C.** DLBCL cell lines were treated with indicated concentrations of cerdulatinib for up to 72 h. Cell numbers were determined at indicated time points and normalized to vehicle treated control. Error bars represent the standard error of the mean (SEM) from three independent experiments.

We next investigated the activity of cerdulatinib in the panel of DLBCL cell lines. A cell-based MTT assay that reflects cellular metabolic activity was performed at 72 h following treatment with cerdulatinib at varying concentrations (Figure 3A). All cell lines demonstrated sensitivity to cerdulatinib with IC_50_ at or below ~2 μM (data compiled in Figure 3B). Notably, ABC cell lines with relatively higher total and phosphorylated STAT3 (Figure 2) displayed good sensitivity to the drug with IC_50_ ranging from 0.29 to 1.80 μM (Figure 3B). Of these ABC cell lines, LY3 and DHL2 were insensitive to a SYK selective inhibitor, PRT060318, as demonstrated in our previous study [13]. LY4, a GCB cell line, was also resistant to the selective SYK inhibition due to lack of surface immunoglobulins [13]. The cell line was, however, sensitive to dual inhibition by cerdulatinib with an IC_50_ of 2.1 μM. Early clinical results suggest that these concentrations can be safely achieved in the plasma of treated patients (Flinn I, et al. 2014 ASCO annual meeting Abstract #2619). Overall, the results demonstrate potent and broad activity of cerdulatinib in DLBCL cell lines. Sensitivity to cerdulatinib was confirmed in an independent cell growth assay (Figure 3C) utilizing two GCB (SUDHL6 and LY18) and three ABC (HBL1, SUDHL2 and LY3) cell lines, which were characterized further below.

### Cerdulatinib induces apoptosis in both GCB and ABC subtypes of DLBCL cell lines via caspase 3 and PARP cleavage

**Figure 4:**
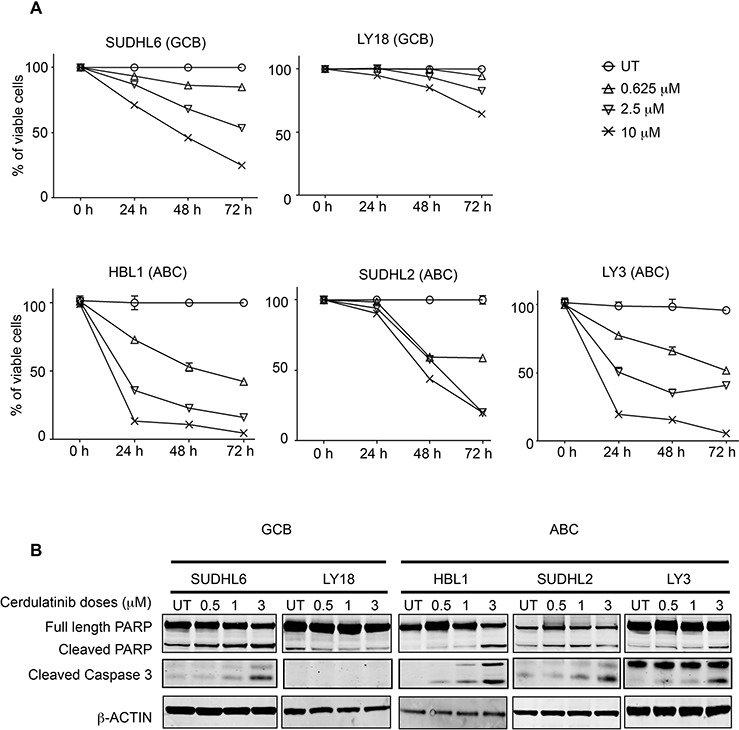
Cerdulatinib induces apoptosis in both GCB and ABC subtypes of DLBCL cell lines via caspase 3 and PARP cleavage **A.** Viability of DLBCL cells treated with various doses of cerdulatinib for up to 72 h. Cells were collected at indicated time points, and stained with annexin V and 7-AAD. Cell viability was measured using flow cytometry, and shown as the percent viable cells (annexin V-/7-AAD-) relative to vehicle treated control. Error bars represent the SEM from three independent experiments. **B.** DLBCL cells were treated with indicated doses of cerdulatinib for 48 h. Western blotting was performed using antibodies against PARP and caspase 3. β-actin was included as a normalization control.

The effects of cerdulatinib on cellular metabolism (MTT) can result from either cell death or cell cycle inhibition or both. We therefore analyzed the effects of cerdulatinib on DLBCL cell viability. Using annexin V and 7-AAD double staining, we demonstrated that with the exception of LY18, viability of all cell lines was reduced by cerdulatinib treatment in a concentration- and time-dependent manner (Figure 4A). Apoptosis induction with increasing concentrations of cerdulatinib in the four responding cell lines was accompanied by both PARP and caspase 3 cleavage whereas little changes were observed in LY18 (Figure 4B).

### Cell cycle arrest by cerdulatinib is associated with inhibition of RB phosphorylation and down-regulation of cyclin E

**Figure 5:**
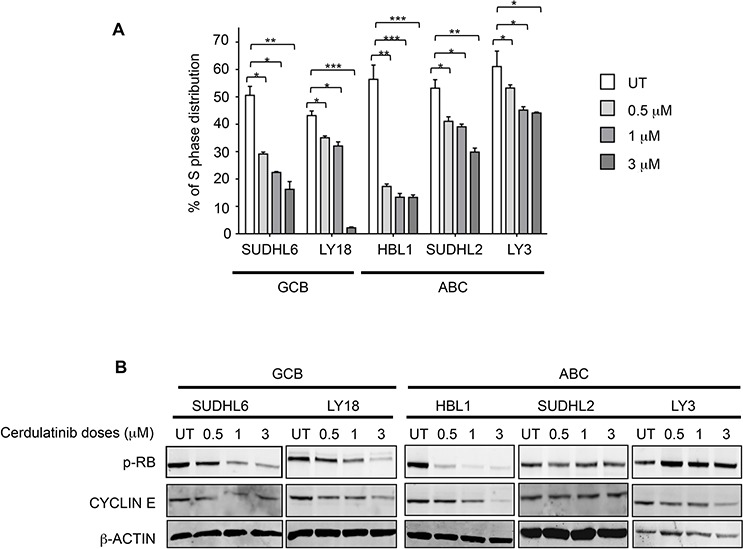
Cerdulatinib blocks cell cycle in both ABC and GCB subtypes of DLBCL via inhibition of RB phosphorylation and down-regulation of cyclin E **A.** DLBCL cells were treated with indicated doses of cerdulatinib for 48 h. Cells were labeled with 10 μM BrdU for 2 h, followed by double staining with BrdU antibody and 7-AAD prior to flow cytometry analysis. Percentage of cells at S phase was statistically analyzed using one-way ANOVA test and graphed using prism 5 GraphPad. Error bars represent the SEM from three independent experiments. *p < 0.05; **p < 0.01; ***p < 0.005. **B.** DLBCL cells were treated with indicated concentrations of cerdulatinib. The whole cell lysates were prepared at 48 h following treatment. Immunoblotting was performed using p-RB and cyclin E antibodies. β-actin was included as a loading control.

After establishing apoptosis induction as one of the drug's actions, we then studied the effect of cerdulatinib on cell cycle progression using BrdU incorporation. As shown in Figure 5A, DHL6, LY18 and HBL1 appeared to be particularly sensitive to cerdulatinib treatment with significant dose-dependent reduction in S phase fraction, while the effects of the drug on DHL2 and LY3 were modest in comparison. The dose-dependent S-phase reductions caused by the drug are largely consistent with its inhibition of phosphorylated RB and with decrease in cyclin E expression (Figure 5B): While bigger changes were observed in HBL1, SUDHL6 and LY18, little or no changes were observed in SUDHL2 and LY3. Collectively, our data on apoptosis (Figure 4) and on cell cycle progression (Figure 5) suggests that cerdulatinib achieves its inhibitory effects in different DLBCL cell lines (Figure 3A and 3B, MTT assay) via different cellular processes, either induction of apoptosis or induction of cell cycle arrest.

### Cerdulatinib induces apoptosis and cell cycle arrest in BCR-stimulated DLBCL cells

**Figure 6:**
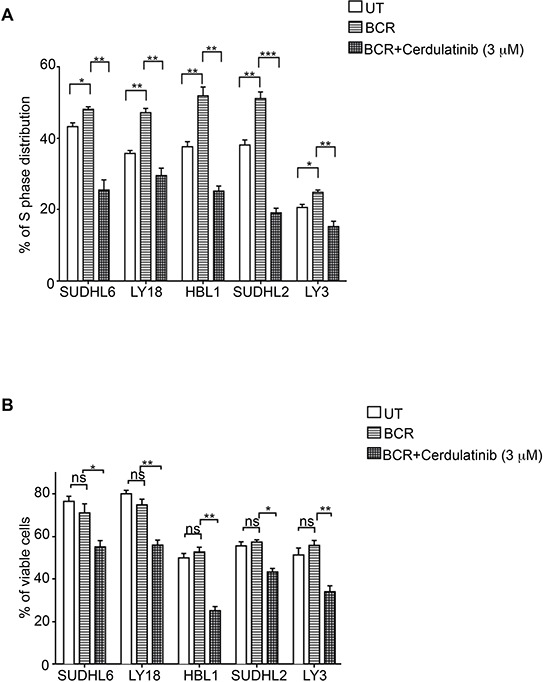
Cerdulatinib induces cell cycle arrest and apoptosis under the condition of BCR stimulation in all DLBCL cell lines **A.** DLBCL cells were treated with 3 μM of cerdulatinib for 48 h and labeled with 10 μM BrdU for 2 h, followed by double staining with BrdU antibody and 7-AAD prior to flow cytometry analysis. **B.** Following 48 hr drug treatment, cells were stained with annexin V and 7-AAD. Percentage of viable cell relative to vehicle control or cells at S phase was statistically analyzed using one-way ANOVA test and graphed using prism 5 GraphPad. Error bars represent the SEM from three independent experiments. *p < 0.05; **p < 0.01; ***p < 0.001.

Since the BCR pathway may be chronically active in many DLBCL, we next examined the capability of cerdulatinib to inhibit cell cycle and induce apoptosis under the condition of BCR stimulation. Figure 6A shows that BCR stimulation with anti-IgM and anti-IgG drove more cells into S-phase in all five cell lines regardless of subtypes and these stimulated tumor cells were sensitive to cerdulatinib treatment. Similarly, the viability of stimulated DLBCL cells were reduced by cerdulatinib in all cell lines tested (Figure 6B). Taken together with the results under the resting conditions (Figures 4A and 5A), we conclude that cerdulatinib achieves its anti-tumor effects in ABC and GCB DLBCL cell lines via induction of apoptosis and cell cycle arrest with or without external stimulation.

### Cerdulatinib blocks JAK/STAT and BCR signaling in both ABC and GCB DLBCL cell lines

**Figure 7:**
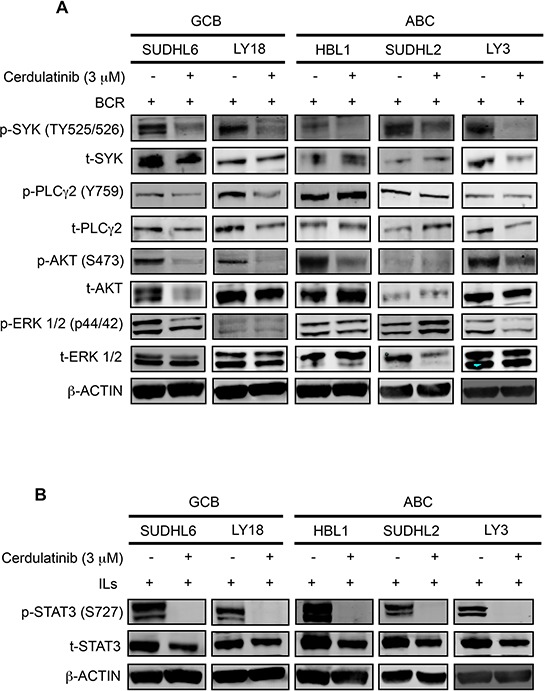
Cerdulatinib blocks JAK/STAT and BCR signaling in both ABC and GCB DLBCL cell lines DLBCL cells were treated with 3 μM of cerdulatinib for 30 min, and then stimulated with either anti IgM/IgG or IL6/IL10 for 24 h before cell collection and lysate preparation. Immunoblotting analysis of **A.** p-SYK, p-PLCγ2, p-AKT, p-ERK expressions and **B.** p-STAT3 expression in selected DLBCL cell lines. β-actin was included as a loading control.

To determine whether cerdulatinib inhibits BCR and JAK-STAT signaling pathways, we first measured the phosphorylation of SYK, PLCγ2, AKT and ERK in BCR-stimulated DLBCL cells treated with or without cerdulatinib (Figure 7A). Immunoblotting analyses revealed a significant reduction of p-SYK in all cell lines, reduction of p-PLCγ2 in the two GCB cell lines (DHL6 and LY18), reduction of p-AKT in four of the five cell lines (except DHL2) and reduction of p-ERK in two of the five cell lines (DHL6 and LY3). When analyzing data by individual cell lines, reduction in protein phosphorylation was observed: SYK, PLCγ2, AKT and ERK in DHL6; SYK, PLCγ2 and AKT in LY18; SYK and AKT in HBL1, SYK in DHL2; and SYK, AKT and ERK in LY3. Thus, despite the variability, at least one signaling component of the BCR pathway was effectively inhibited by cerdulatinib in each individual cell line.

We also determined whether cerdulatinib inhibits the JAK-STAT signaling pathways under the condition of cytokine stimulation. Cells stimulated with IL-6 and IL-10 were treated with cerdulatinib or vehicle, phosphorylation of STAT3 at Y705 was measured. As shown in Figure 7B, cytokine stimulated cells expressed very high levels of p-STAT3 regardless of subtype designation. Addition of cerdulatinib completely blocked this phosphorylation event to a level that goes below the detection level. Thus, we conclude that modulation of both BCR and JAK-STAT signaling pathways by cerdulatinib correlated with its cellular toxicity in terms of cell survival and proliferation (Figure 6).

### Cerdulatinib induces cell death in primary human DLBCL samples

**Figure 8:**
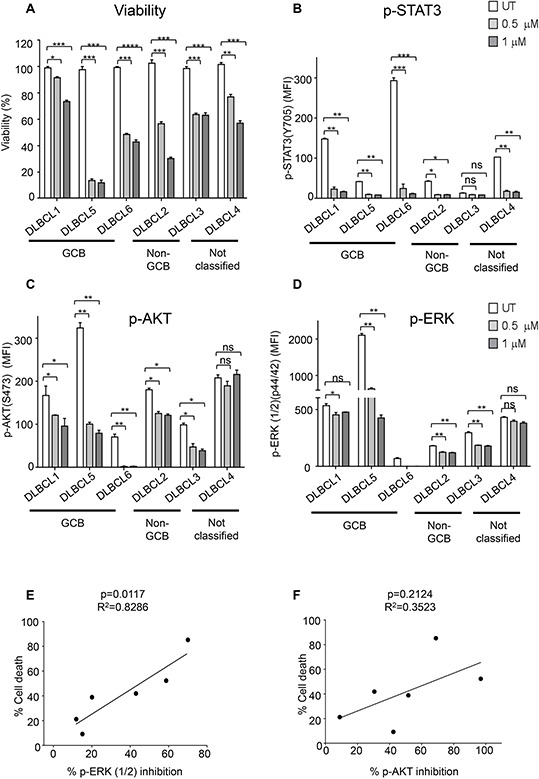
Primary DLBCL cells are sensitive to dual SYK/JAK inhibition **A.** Six primary human DLBCL samples were treated with 0.5 μM or 1 μM cerdulatinib for 72 h. Cell viability was measured by MTT assay, and normalized to vehicle control. Primary human DLBCL samples were treated with 0.5 μM or 1 μM cerdulatinib for 6 h followed by stimulation with 5 μg/ml IgM/IgG for 15 min at 37°C. Phospho-flow assays were performed to determine the levels of p-STAT3 in **B**, p-AKT in **C** and p-ERK in **D** in the primary cells. Error bars represent the SEM from three independent experiments. *p < 0.05, **p < 0.01, ***p < 0.001, ****p < 0.0001. **E.** Relationship between cell death following treatment with 1 μM cerdulatinib and percent inhibition of ERK1/2 was analyzed by Spearman correlation. **F.** Relationship between cell death and percentage of inhibition of AKT was statistically analyzed using one-way ANOVA test. Percent inhibition of phosphorylation was calculated using the formula: [(MFI_untreated_-MFI_treated_)/MFI_untreated_] × 100%.

To confirm whether data obtained with the cell lines are reproducible with primary patient tumors, we tested several human DLBCL samples, including 3 GCB, 2 non-GCB and one unclassified (patient information shown in Supplemental Table 1), for their apoptotic response to 0.5 μM and 1 μM cerdulatinib. As shown in Figure 8A, all primary DLBCL cells responded to cerdulatinib in a concentration-dependent manner with variable sensitivities.

We next studied the inhibition of p-STAT3, p-ERK and p-AKT in response to cerdulatinib treatment in these primary patient cells stimulated with anti-BCR. Phospho-flow assay demonstrated a marked and simultaneous down-regulation of p-STAT3, p-AKT and p-ERK in response to increasing concentrations of cerdulatinib in all six primary DLBCL cells regardless of their subtype designation (Figures 8B, 8C and 8D), but inhibition of the individual protein phosphorylation was highly variable among these primary samples. For instance, DLBCL3 showed no significant inhibition in p-STAT3 in response to increasing concentrations of cerdulatinib, presumably due to its very low baseline p-STAT3 level in untreated cells compared to the other patient samples (Figure 8B). DLBCL1 and DLBCL4 had little to no suppression of p-ERK activity upon cerdulatinib treatment (Figure 8D). These results are similar to those observed in cell lines (Figure 7) suggesting individual patient samples may rely upon different signaling pathways. Interestingly, a significant linear correlation was found between the degree of p-ERK inhibition and the extent of cell death response (Figure 8E, left, *p* = 0.0117). Meanwhile, no significant linear correlation was identified between the extent of p-AKT inhibition and the cell death response (Figure 8F, right, *p* = 0.2142). The results suggest that indirect inhibition of downstream ERK phosphorylation by cerdulatinib may play a role in the final cellular outcome, and ERK inhibition may mediate the therapeutic effects of cerdulatinib.

## DISCUSSION

Available clinical data indicate that BCR-directed inhibitors such as SYK (fostmatinib), BTK (ibrutinib) and PKCβ (enzastaurin) are active in ≤ 20% of DLBCL patients. This highlights the need to continue exploring novel agents with expanded activities in this disease. In this study, we first demonstrated the presence of active SYK and JAK in their phosphorylated forms in a significant fraction of DLBCL primary tumor tissues and cell lines. We then showed that cerdulatinib, a potent inhibitor of SYK and JAK, has broad anti-tumor activity as demonstrated by inhibition of 1) cellular metabolic function, 2) cell viability, 3) cell cycling, 4) signal transduction through SYK-PLCγ2-AKT or ERK, and 5) signal transduction through JAK-STAT. We further demonstrated that cerdulatinib induced cell death in primary DLBCL cells and the degree of cell death significantly correlated with decreased p-ERK.

While the broad activity of cerdulatinib in DLBCL cell lines may be ascribed to mechanisms beyond SYK and JAK inhibition (Table 1), the data presented herein with DLBCL cell lines and primary tumors are at least consistent with the hypothesis that SYK and JAK inhibition contributes to the antitumor activities of the drug. Target selectivity of cerdulatinib was demonstrated previously in different cell types of normal human whole blood as well. The compound potently inhibit BCR and FcR-induced SYK activation and cytokine receptor-induced JAK1/3 and JAK1/TYK2 activation in B cells, T cells and monocytes while it does not inhibit protein kinase C-mediated PMA signaling or LCK and ZAP70–mediated T cell antigen receptor signaling, or JAK2-mediated GM-CSF signaling. [26]

Cerdulatinib demonstrated broader anti-tumor activity relative to several BCR-specific inhibitors we have evaluated. Previously, we have shown that dasatinib (targets mainly SRC family kinases at low concentrations) and PRT318 (highly specific for SYK) exert anti-tumor activity primarily by affecting cell cycle with minimal impact on cell viability [12, 13]. However, with cerdulatinib, we observed apoptosis induction in addition to cell cycle inhibition (Figures 4–6) although the specific cellular effect varied from cell line to cell line. These data suggest that simultaneous inhibition of multiple therapeutically relevant targets, such as combined SYK and JAK, may represent a more effective approach compared to single target-directed agent.

It is also noteworthy that many cell lines resistant to BCR-targeted inhibitors were sensitive to cerdulatinib. Among the cell lines studied, LY3 carries an activating mutation in CARD11 [31] (Supplemental Table 2); LY3, LY10, U2932 (ABC) along with LY8, VAL, and SUDHL2 (GCB) carry inactivating mutations or hemizygous deletion in *TNFAIP3 (A20)*, which negatively regulates NFκB activity. Loss of *A20* function results in constitutive activation of NFκB that promotes tumor growth and survival. ABC cell lines, HBL1, LY3, LY10 and SUDHL2, along with GCB cell line SUDHL6 all carry activating MYD88 mutations, which promote IRAK1/4 and TRAF6 dimerization and subsequent NFκB activation.

The ultimate activation of NFκB, resulting from these BCR-associated or MYD88 mutations, leads to increased IL6 and IL10 autocrine secretion that subsequently activates JAK-STAT3 pathway to enhance cell survival and proliferation. This perhaps represents one of several mechanisms rendering tumor cells resistant to inhibitors of early BCR components. As a matter of fact, high levels of baseline phosphorylated STAT3 exhibited in the ABC cell lines (Figure 2) may be a result of the BCR/MYD88 mutations they carry. Cerdulatinib, with its ability to inhibit p-STAT3 in both cell lines and primary tumors, provides another point of blockade after NFκB activation. Antitumor activities were achieved by cerdulatinib in all cell lines carrying mutations including ABC cell lines HBL1 (MYD88), LY3 (CARD11, A20 and MYD88), LY10 (A20 and MYD88), SUDHL2 (A20 and MYD88), and U2932 (A20) as well as GCB cell lines LY8 (A20), SUDHL6 (MYD88) and VAL (A20) (Supplemental Table 2), likely by blocking the up-regulation of STAT3 activity induced by the sequential events of BCR/MYD88 mutations—NF-κB activation—cytokine release-JAK/STAT. In particular, not only high levels of baseline phosphorylated STAT3 exhibited in the ABC cell lines render these cells very sensitive to cerdulatinib inhibition, but also cytokine-induced STAT3 phosphorylation in GCB cell lines (Figure 7B). Notably, some of these cell lines, LY3 in particular, was shown to be highly resistant to inhibition of SRC, SYK and BTK [10]. In the clinical trial setting, patients with mutations in CARD11, A20 and MYD88 indeed demonstrated resistance to ibrutinib, a BTK inhibitor [17]. In addition to BCR inhibitor-resistant DLBCL, cerdulatinib (PRT2070) also has the capability to overcome ibrutinib resistance in CLL cells carrying BTK^C481S^ mutation [28, 29]. These data suggest that simultaneous inhibition of multiple therapeutically relevant targets, such as combined SYK and JAK, may represent a more effective approach for the treatment of B-cell lymphoma.

## MATERIALS AND METHODS

### Cell lines, primary cells and culture conditions

GCB cell lines OCI-LY1, OCI-LY4, OCI-LY8, OCI-LY18, SUDHL6 and VAL were cultured in Iscove's Modified Dulbecco's Medium (IMDM) supplemented with 20% Fetal Bovine Serum (FBS) and 100 μg/mL penicillin/streptomycin. ABC cell lines SUDHL2 and HBL1 were cultured in RPMI1640 supplemented with 20% FBS and 100 μg/mL penicillin/streptomycin. OCI-LY3 cells were grown in RPMI1640 supplemented with 20% FBS, 100 μg/ml penicillin/streptomycin and HEPES. ABC cell lines OCI-LY10 was cultured in Iscove's Modified Dulbecco's Medium (IMDM) supplemented with 20% human serum and 100 μg/mL penicillin/streptomycin. ABC cell line U2932 was cultured in high glucose RPMI1640 supplemented with 20% FBS, 100 μg/ml penicillin/streptomycin. All cells were maintained in a humidified 37°C/5% CO_2_ incubator. Frozen primary DLBCL cells were obtained from the tumor bank of the Department of Pathology at Weill Cornell Medical College after Institutional Review Board review and approval. All primary cells were thawed as previously described [32] and cultured in above conditions at 37°C for 6 h in the presence or absence of cerdulatinib followed by BCR stimulations with 5 μg/mL of IgM and IgG. The clinical characteristics of these patients are listed in the Supplemental Table 1. Cerdulatinib [26] was kindly provided by Portola Pharmaceuticals Inc. (South San Francisco, CA) and stored as 10 mM stock at −20°C.

### Immunohistochemistry

The study included 62 DLBCL samples in two tissue microarrays from 2000–2011. Twenty-five cases were from nodal sites and 37 from extranodal sites. Only tumors with enough tissues were included. Immunohistochemical stains of p-STAT3 (Y705, rabbit monoclonal Ab, clone D3A7, Cell Signaling) and p-SYK (Y525/526; rabbit polyclonal Ab; Cell Signaling) were performed and the results were evaluated by proportion of lymphoma cells that were stained. The antibody reactivity in ≥ 30% of the lymphoma cells was considered positive. Paraffin-embedded normal tonsil tissue was included as a baseline expression level for p-STAT3 and p-SYK.

### Immunoblotting assays and antibodies

Immunoblotting were performed as previously described [32]. Antibodies including total STAT3, SYK, PLCγ2, AKT, ERK1/2, PARP, caspase 3, and phosphorylated STAT3 (S727), STAT3 (Y705), SYK (Y525/526), PLCγ2 (Y759), PLCγ2 (Y1217), AKT (S473), ERK1/2 (Y204) and RB (S807/811) were purchased from Cell Signaling (Danvers, MA). Cyclin E antibody was purchased from Santa Cruz Biotechnology (Dallas, TX), and β-actin antibody was purchased from Sigma-Aldrich (St Louis, MO). Antibodies used in phosphoflow assay, including phosphorylated STAT3 (S727), SYK (Y525/526), PLCγ2 (Y759), AKT (S473), and ERK1/2 (Y204) were purchased from BD Biosciences (San Jose, CA).

### Cell metabolic activity, cell growth and viability determination

DLBCL cell lines were treated with various concentrations of cerdulatinib for up to 72 h. The metabolic activities of cells were determined with MTT assay according to manufacturer's instruction (Roche Applied Science, Indianapolis, IN) at 72 h time point. IC_50_ was calculated using the Sigma Plot generated with GraphPad Prism 6 software (GraphPad, La Jolla, CA). Cell growth was measured at every 24 h counting live cells with flow cytometry as previously described [12]. Cells were collected every 24 h and cell viability was determined by the PE Annexin V Apoptosis Detection Kit I (BD Biosciences).

### Cell cycle analysis

DLBCL cells were treated with various concentrations of cerdulatinib for 48 h. Cells were incubated with 10 μM BrdU (BD Biosciences, San Jose, CA) at 37°C for 2 h, and stained with PE conjugated anti-BrdU antibody (BD Biosciences) according to the supplier's manual. The percentage of cell cycle distribution was analyzed with FlowJo (Tree Star Inc. Ashland, OR).

### Intracellular phosphospecific flow cytometry assays

DLBCL cells were treated with cerdulatinib for 6 h followed by stimulation with 5 μg/mL of goat F (ab')2 anti-human IgM and IgG antibodies (Southern Biotech, Birmingham, AL) at 37°C for 15 min. Cells were fixed in 4% formaldehyde at room temperature for 10 min, and permeabilized with 100% methanol on ice for 20 min before flow cytometric analyses.

### Statistical analyses

A One-way ANOVA test was performed to compare the percentage of S phase distribution or the MFI fold changes between the treated and untreated DLBCL cell lines/primary cells. The relationships between the percentage of cell death response and inhibition of ERK1/2 or AKT phosphorylation were analyzed by Spearman correlation

## SUPPLEMENTARY TABLES


