# Supplementary material for: Cerdulatinib, a novel dual SYK/JAK kinase inhibitor, has broad anti-tumor activity in both ABC and GCB types of diffuse large B cell lymphoma
Source: Oncotarget. 2015 Nov 5;6(41):43881–96. doi: 10.18632/oncotarget.6316 (PMC4791274; doi:10.18632/oncotarget.6316)
Supplement: Supplementary file 1 [file oncotarget-06-43881-s001.pdf]

SUPPLEMENTARY TABLES

Supplementary Table S1: Immunohistochemical features of primary DLBCL tumors

Patient ID	Age/Sex	Specimen Type	CD10	BCL6	BCL-2	MUM-1	CD20
DLBCL1	70/M	Left Groin LN	> 90%	> 60%	> 90%	60–70%	90%
DLBCL2	68/F	Left Axillary LN	< 5%	< 5%	70–80%	60–70%	80–90%
DLBCL3	59/M	Right Axillary LN	< 1%	+	+	+	89%
DLBCL4	67/F	Right Axillary LN	6%	N/A	N/A	N/A	90%
DLBCL5	61/F	Right inguinal LN	95%	70%	95%	80%	90%
DLBCL6	49/F	Right Colon & Terminal Ileum	93%	N/A	N/A	N/A	96%

Abbreviations: M, male; F, female; N/A, not available. LN: lymph node. BCL6, BCL2 and MUM-1 were determined by immunohistochemical staining and CD10, CD20 was determined by flow cytometry. Tumors content of the primary cells were determined by flow cytometry of CD20-stained cells.

Supplementary Table S2: Common gene mutations in DLBCL cell lines

Sample ID	Subtypes	CARD11	EZH2	MYD88	TNFAIP3/A20
LY1	GCB	WT	m	WT	WT
LY4	GCB	WT	WT	WT	WT
LY8	GCB	NK	WT	WT	del
LY18	GCB	NK	WT	WT	NK
SUDHL6	GCB	WT	m	m	WT
VAL	GCB	WT	m	WT	m
HBL1	ABC	WT	WT	m	WT
LY3	ABC	m	WT	m	del
LY10	ABC	WT	WT	M	del
SUDHL2	ABC	WT	WT	m	M/M
U2932	ABC	WT	WT	WT	del

WT, wild type; NK, not known; m, missense mutation; M/M, biallelic truncating mutation; del: hemizygous deletion. Adapted from [31].
